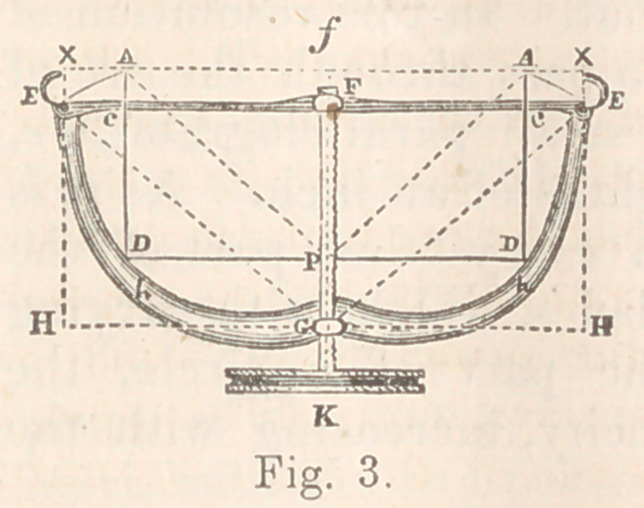# A Spring and Self-Retaining Speculum

**Published:** 1868-03

**Authors:** Nathan Bozeman

**Affiliations:** Fifth Avenue Hotel; New York


### A SPRING AND SELF-RETAINING SPECULUM.

By NATHAN BOZEMAN, M.D., New York.

The vagina, as a membranous canal, in the distended state, may properly be said to represent a truncated cone with the base turned upward and the apex downward, corresponding with its mouth.

The general outline of the organ, as viewed in its natural condition, is such as would result from bringing the two opposing walls of the cone together, the cervix uteri being encircled by it at the centre of its base, and its mouth closed by the falling together of the labia majora.

The line, therefore, formed by the anterior and posterior walls of the organ coming together is transverse, while that formed by the opposing surfaces of the labia is antero-posterior, being at right angles.

Now the most natural indications for the dilatation of this canal with the peculiarities named would appear to be, first, separation of the labia, and, second, the two opposing walls of the collapsed cone, so to speak. This, scarcely need I say, is the view generally taken of the relationship of these parts, and the usual practice is based upon it of bringing within the field of observation the cervix uteri and the two vaginal walls.

This plan of antero-posterior dilatation of the vagina, it matters not what form of speculum is used, I conceive to be a popular error, and it is wholly at variance with the true anatomical relation of the parts. I shall, presently, attempt to explain more fully my meaning in our description of *a new form of speculum*, which I have the pleasure of presenting now to the notice of the profession. The principle of construction, as well as principle of action of this new instrument, will be found to differ from all others heretofore in use, in several respects, which I shall explain farther on. Suffice it to say, one of the very essential differences is in what might be termed the working point of the instrument, that portion which is applied to the resistance. The blades of our instrument are introduced between the opposing walls of the vagina edgewise, instead of flatwise, as formerly; and the dilatation is effected transversely or horizontally, as will be better understood when we come to explain the principle of action. The same instrument applies to the dilatation of the vulva as well as the vaginal canal; thus giving us, at one glance, a view of the parts from the mons veneris to the cervix uteri in front, and behind, nearly the whole of the posterior wall of the vagina—any and every point within this extensive range being accessible for operative purposes.

The dilatation thus effected is so regulated that the labia and the two extremities of the vagina are put upon the stretch only to the extent desired, which is in strict accordance with the anatomical conformation of the parts, this being of such a nature as to make the instrument *self-sustaining*, one of its peculiarities; another being *elasticity of flexure*. This principle of elasticity has never before been embodied in any form of speculum that I am aware of, and its utility and importance, in my judgment, cannot be too highly estimated. Instead of the hard, inflexible blade formerly used, touching only at one or two points soft and delicate structures, we have now the soft, elastic spring adapting itself to all the points of resistance with a uniformity to be attained in no other way.

The indications for complete dilatation of the vagina and vulva I conceive to be four:

1st. Elevation of the perinæum.

2d. Elevation and support of the upper part of the posterior wall of the vagina.

3d. Transverse dilatation of the labia majora and the mouth of the vagina.

4th. Distension and steadiness of the upper part of the anterior wall of the vagina, the vesico-vaginal septum.

Now these are the indications to be fulfilled, according to my judgment, independent of any and all efforts of the patient to the contrary; and any instrument, whether *self-retaining or not*, that does not meet these ends, must be regarded as incomplete. With my instrument, I claim the accomplishment of all, *the fulfilment of the third and fourth indications* being an advance beyond all other methods, to say nothing of the *self-retaining* quality of the instrument, which, it must be admitted, is based upon more correct principles than any plan heretofore presented to the notice of the profession.

As regards the position of the patient, I propose a few remarks before entering upon the description of our instrument, as I consider this of no little consequence in certain operations, especially those upon the anterior wall of the vagina.

While our speculum is equally well adapted to all positions,

I prefer, in the description and application of it, to consider the patient resting upon her knees and breast, the body forming a right angle with the thighs, and the thighs a right angle with the legs. This position I now prefer to all others, and, with propriety, it may be termed the *right angle position upon the knees.*

In no other position, according to my judgment, whether chloroform be used or not, can the patient be made so easy, comfortable, and secure, and without the aid of assistants. Our supporting frame, when folded up, is compact, light, and portable, and weighs only eleven pounds. It exceeds twelve inches in height, only on one side, the depth and width being twelve by eighteen inches. I hope before long to be able to publish a description of this *thoracic rest* or *support.*

We have come now to the most difficult part of our task, a description of this speculum.

Fig. 1 (half size) represents a front quarter view of the instrument, expanded, as when introduced for use.

The general features of it, as shown, are outstretched arms, expanded wings, rolling surfaces, standing and projecting arches, broad, contracted, narrow, and rounded points; and the thumb-screw arrangement indicates that the whole is moved by a system of leverage.

*Fig. 1*

The proportions of the instrument, are, I think, in harmony, and the construction will be found to be in strict accordance with well-known geometrical principles. It may be said to be composed of two simple, similar bent steel levers, about $8\frac{1}{2}$ inches in length, rounded and flattened at certain points, having elasticity of flexure, and connected at one extremity by a pivot-joint, around which they revolve horizontally.

For description, therefore, as is most naturally suggested from its general outline, it may properly be divided into the foot and heel, including thumb-screw and short levers, and into the legs, body, wings, neck, and arms or blades, as indicated by Figs. 1, 2, 3, 4, and 5.

The description of the foot and heel we will defer until we come to study the principle of action.

I shall consider B the centre of the instrument; the plumb line U, dropped from it, the balancing-point.

The legs, where they leave the heel E and *e* are rounded, a-quarter of an inch in thickness, and for a short distance ascend almost perpendicularly, inclining slightly forward and inward. In the next part of their course they become gradually more and more flattened, extending now almost directly forward, only inclining slightly outward.

The line U indicates their union with the body. Their length is two inches and three-eighths. This part of the instrument applies to the purpose of dilating the vulva or labia majora. The lower part of the legs falls just within the fold formed by the inner part of the thigh and the labia, while the upper portion passes between the latter about the commencement of the nymphæ, and thus reaches the mouth of the vagina, which corresponds exactly with the plumb line U, the balancing-point.

The body is included between the two lines U and Q, and is somewhat quadrangular in shape, rounding on its outer surface, and hollowed out on the inner side to the same extent as the upper part of the leg and the wing standing upon its upper edge, as indicated by the line B Q. This part of the instrument is applied directly to the transverse dilatation of the mouth of the vagina. The wing is of a peculiar shape, and, for the sake of description, may be divided into the lower and inner portion, and the upper and outer portion. The first part presents a rounded surface from right to left, and up toward the projecting angles R *r* looks almost directly forward. These projecting arches are about three-quarters of an inch wide, and at the angles are about one inch above a line drawn across from centre to centre. This part of the wing, with its fellow of the opposite side, gives support to the perinæum, which lies across from one to the other, just as the bridge spans the stream.

The upper and outer portion of the wing looks forward and outward, and is intended to support the buttock. The neck, between the two plumb lines Q, L, is about half an inch in length and width, and, as shown, is the most contracted part of the arms. This point comes just within the mouth of the vagina, and, consequently, prevents painful stretching of the parts here in the expansion of the blades.

The arms or blades form the widest part of the instrument, and are intended to distend and steady the vesico-vaginal septum. They are thin, spoon-shaped, about two inches and three-eighths in length, and at M one and a-half inches wide. On the middle of this line is seen the countersunk head of the rivet which passes through here and gives support on the inside to the extremities of the arch N *n*, connecting the blades at this point. This arch is four and a-half inches in length, connected in its middle by a hinge-joint O, and about three-quarters of an inch in width. It should be made of steel, and so thin between the joint and extremities as to allow of easy bending in the opening and shutting of the arms. There are two holes near each end, with slits in the upper edge, to encircle the narrow neck of the rivet when in use. This arch may be used or not, as circumstances may require, it being easily slipped off or on. When used, it is intended to elevate and support the upper part of the posterior wall of the vagina, it being the fulfilment of our second indication. It is easily elevated or depressed with the finger, and when in position stands about one inch above the edges of the blades, and on a plane slightly above that of the projecting angles of the wings R *r*. Nearly the whole of the instrument, as will be seen by reference to the figure, is included within the legs of the right-angled triangle E B C, only the foot, legs, and wings being outside. The circle D R Q S has its centre at B, the centre of the instrument, with a radius of one inch and a-quarter, the length of the line of union between the root of the wing and the body. This circle, as is seen, includes nearly the whole of the wing, the body, and a large part of the leg. This angle and centre of circle, I should observe, are important points to be borne in mind in the manufacture of the instrument. They should be preserved in all cases, it matters not what change may be found necessary, as regards proportions.

The instrument, when set upon a table, has its foot flat upon the surface, touching nowhere else excepting at the point near the ends of the blades, as indicated by the base line of the angle E C, which measures four inches and three-quarters.

The leg E B measures two inches and three-quarters, and C B four inches and a-half.

From centre B to corresponding point of opposite side, the distance is two inches and a-quarter. Between tips of wings D *d*, four inches and a-quarter. Between commencement of neck Q, three inches. Between blades at M, measuring from outside to outside, four inches. Between points, measured in the same way, three inches and a-half.

The instrument is to be made of steel, electro-plated, as light as is consistent with the strength required, there being certain points, of course, where this is an important feature; for example, the foot and heel of the instrument.

The *elasticity of flexure*, it should be borne in mind, extends only from the heel to the extremities of the blades, increasing, of course, in extent as the latter points are approached. The limit of elasticity at the points of the blades should not exceed three-quarters of an inch under any amount of resistance here, and this should be borne in mind in *tempering* the instrument, otherwise the limit might be exceeded, and the usefulness of the instrument thereby endangered.

Fig. 2 represents the instrument closed, ready for introduction or withdrawal. It being collapsed, so to speak, every point of the opposing sides is brought into closer relationship. The elevated arch standing above the edges of the arms or blades, as seen in the first view, is now folded within them, the upper part of it resting beneath the hugging arches, R *r*.

*Fig. 2*

In this view of the instrument, there are three divisions made by the two plumb lines U and Q, which are important, as directing attention to the uses of the respective portions. The leg, for example, included within the first division, performs the part of separating the *labia majora*. The wings and body of the second division elevate the *perinœum*, and open the *mouth of the vagina*, to the utmost limit transversely. The arms or blades of the third division *unfold* and *steady* the *vesico-vaginal septum*, or upper part of the anterior wall of the vagina, and at the same time give support to the two extremities of the arch which spans the space between them, and receives upon its top *the falling posterior wall of the vagina.*

The thumb-screw K is seen reversed to its fullest extent, and the two short levers quietly folded within the foot of the instrument, the point P being now in close proximity to the pivot G.

We come now to a consideration of the principle of the instrument, and, I will state in the outset, as thus applied, it is new and original with myself, it never having been applied before, that I am aware of, by any one, to the purposes of a speculum.

The principle itself, however, is an old one, as regards its application to other purposes. It will be familiar to those who may have seen a certain kind of *cotton press* in the Southern States, in which it is employed, though with a more extensive system of leverage than I have here. I got the idea myself from seeing the above application; and the credit I am entitled to is the modification which I have made of it, to suit the purposes of a *self-retaining* speculum, the principle of which we will now attempt to describe. This principle, as here applied, I have no hesitancy in saying, forms one of the most beautiful illustrations of the parallelogram of forces as producing curvilinear motion that could be conceived, and answers, in the most satisfactory manner, the purpose for which it is here intended.

In studying the law of forces, there are several important points always to be borne in mind, whether applied to the rudest lever or pulley, or to the most complex piece of machinery. As these points are or are not understood, depends success or failure.

Professor Silliman,* who is authority in matters of this sort, says: "To determine a force with precision, we must consider three things: 1st. The point of application. 2d. The direction. 3d. The intensity or energy with which the force acts."

Inattention to one or more of these rules has, I am satisfied, caused the failure of all previous efforts at getting up a *self-*

* Principles of Physic.

*retaining speculum*, to fulfil all the indications previously named. I am free to confess myself that I failed in many of my efforts from this very cause.

My greatest error I now conceive to have been in the point selected for the application of force. Had I the time and space, it might be interesting to show how I labored to extricate myself from this difficulty; but, as it is, I shall be content for the present with saying that this instrument, as here exhibited, is not the work of a day, or a week, or a month, but years of patient thought and repeated disappointments.

Let us now turn our attention to the diagram, Fig. 3, which is also a half-size front view of what I have denominated the foot of the instrument, here represented closed and expanded, with both legs cut off at the heel E and E.

The two sides E *h* G together form, as is seen, almost a semicircle, with a radius of one and a-quarter inches. In the middle, where they unite, is the pivot-joint G, and here is the point of our application of force.

These arms are inflexible, somewhat round, and almost of uniform thickness, not exceeding a quarter of an inch anywhere, excepting at the pivot and ends, where they swell out a little, to give additional strength.

Fig. 3.

Within these arms is situated our plan of leverage for opening and shutting the instrument. This consists of a double-threaded thumb-screw K, about one inch and three-quarters in length, and three-eighths of an inch in thickness, with an open wheel on the outer end, one inch and an-eighth in diameter; and of two short, stout levers, one and a-quarter inches long. These latter are connected at one extremity by a joint at the heel E and E, two and a-half inches from the pivot G. At the other extremity, they are connected together by a joint at P. Rising above, three-eighths of an inch, is to be seen the connecting screw of this joint, expanded, and perforated to receive the extremity of the thumb-screw, upon the extremity of which, on the outside, is placed a small tap. In the same manner, the thumb-screw passes through the connecting pivot-screw G, which is the nut, the former being free to move both forward and backward.

Let the two lines now on each side, A D and P D, represent the instrument closed, A *f* and P *f* completing the rectangle or square. The diagonal P A will then represent the situation and relationship of the two short levers previously described.

To open or expand these arms now to the full extent, we have, as would appear, two forces, A P and A P, acting at an oblique angle, a very great mechanical disadvantage, as will be readily understood, for, "when a force acts upon a body at any other than a right angle, a part of its effect is lost."

The difficulty, however, is overcome, and the accomplishment of our purpose rendered easy, by resolving each of these oblique forces into two, P *f* and A *f*, one parallel and the other perpendicular to the point to be moved. This is effected by revolving the thumb-screw K until it assumes the position of G F, and the short levers that of E F and E F. The latter together now form a straight line—a relationship that places the whole instrument in a state of equilibrium; the weight of the two sides, being equal, is exactly counterpoised at F. Complete now the parallelogram E H G, and we have the diagonal G E, the resultant of the two components thus applied, which give us the diagonal or oblique relationship of the arms of the instrument which is here so beautifully carried out. In this resolution of forces, therefore, our power is seen to pass through the arc of a circle which is the diagonal of the small parallelogram A *c*, E *x*, the distance A E being three-eighths of an inch. As it is with the seat of power, so it is with every other part of the instrument to the extremity of its blades, which, with varying radii, passes through the arc of the part of a circle, the length of which, as well as the velocity, increasing with the distance from the pivot G.

For instance, at the centre or balancing-point of the instrument, U, Fig. 1, corresponding to the mouth of the vagina and about one inch from the seat of power, we have the arc increased from three-eighths to half an inch, with a total expansion of the arms at this point of two and a-half inches. And at the extremity of the blades, a distance of about five inches from the same point, the arc is increased to one and a-half inches, giving us a space between the opposing blades of three inches for operative purposes.

At the two points named, the limit of expansion of the blades corresponds exactly with the limit of the dilatation of the mouth of the vagina, and its upper extremity, which alone is sufficient to explain the *self-sustaining* and *self-retaining* feature of the instrument.

In the application of our power then to the thumb-screw K, the position of it is most advantageous for producing its maximum effect in collapsing or carrying the two short levers from their oblique relationship to that of right angles with the point acted upon, thus affording an example of increased power with increased resistance. The instrument with the above system of leverage may properly be said to represent a *double bent lever*, the most familiar example of which is the *fire-tongs.* Unlike these, however, it has the power applied on the inside instead of the outside. Alike, though, in the important respect of having the power applied between the fulcrum and the weight or resistance, distinguishing both at once as levers of the third class.

This instrument I shall call the *spring and self-retaining speculum*, as is most naturally suggested from these two distinguishing qualities of it.

I think I may justly claim for this speculum originality in:

1st. The system of leverage employed, possessing, as it does, regulated and increased power, reduced to the smallest possible compass, and far away from the mouth of the vagina, thus allowing the freest and widest range of manipulation with instruments, compatible with the nature of these parts.

2d. Transverse action of the instrument, with uniformly varying movement of the working-point, extending from the heel to the point of the blades, thus making the lateral walls of the vagina the seat of pressure instead of the anterior and posterior, as formerly.

3d. Complete exposure, at the same time, for operative purposes of the vulva, both walls of the vagina, and the cervix uteri, with the two polished surfaces of the arms of the instrument standing upon the sides, the most favorable position in which they could be placed to secure the greatest amount of reflected light.

4th. Elasticity of the working-point of the instrument.

5th. Being self-retaining in the fullest sense of the word.

6th. Being equally applicable in its use to all positions of the patient.

7th. Allowing all operations to be done without the aid of assistants, or exposure of the person of the patient, further than the parts immediately brought within the field of observation by the expansion of the arms of the instrument.

All of these points, I am safe in saying, admit of the clearest demonstration.

*Remarks.*—Having now completed the description of our *spring and self-retaining speculum*, it remains for us to offer a few additional remarks upon its application in practice, and the circumstances under which it was first done; for it is reasonable to conclude that the question will be asked, where is the proof of all the advantages which have been portrayed at such length?

The only proof I propose to offer, and I think this conclusive, is the application of the instrument in a single case, the very one to which it was adapted in completing it as here shown. This case being an extreme one, as will appear, has the advantage, I think, of rendering the proof convincing to the practical mind, and lessens the necessity, I conceive, of additional corroborative testimony to satisfy even the most skeptical. The case referred to was one of vesico-vaginal fistule, occurring in a very stout, fleshy woman, weighing upwards of two hundred pounds. Early in October last, she was admitted into that admirably conducted institution under the direction of the Sisters of the Hoboken St. Mary's Hospital, where my patients are now received.

The fistule was of six or eight months' standing; small, not larger than a pin's head, and occupied what we would ordinarily term a favorable position, being some three inches above the *meatus urinarius*, and near the edge of the septum, upon the left side.

The peculiarity and difficulties of the case were these: Anteversion of the uterus; a convoluted or folded condition of the two opposing walls of the vagina, which was of immense size; and a pleated condition of the edges of the fistule, and the parts immediately surrounding it.

Assisted by Drs. Finnell, Connolly, Lynch, Metcalfe, and several other medical gentlemen of New York, and Dr. Chabert, of Hoboken, I undertook my usual operation, the patient resting upon her knees and elbows. My fourth size of the lever speculum, with a blade four inches long, one and a-half inches wide at the heel, and one and three-quarter inches near the point, was employed; and, although of such large size, this instrument, with spatulas and depressors, brought to bear from various points by assistants, afforded us only an imperfect view of the very small fistule. The upper part of the posterior wall of the vagina came down in such immense folds over the end of the instrument, met by the same folded and protruded condition of the anterior wall, under violent and almost continuous expulsive efforts, that it became quite impossible to commence the process of paring the edges of the fistule, and to complete it in a regular manner. This stage of the operation, however, was gone through with after the length of time indicated, only to be followed by a still greater difficulty and delay in the next —the introduction of our sutures—only three being called for. The patient, at this stage of the operation, was placed upon her side and chloroformed, which, however, afforded us no relief from the surrounding difficulties.

Suffice it to say, the operation, after three hours, with five or six assistants, was finished, though in the most unsatisfactory manner it had ever been my misfortune to encounter before.

Now, after all our labor and annoyance, I felt that a failure was inevitable, and so expressed myself to the gentlemen present. The removal of our suture apparatus, on the eighth day, proved too truly the correctness of our misgivings as to the final result. There was a total failure.

With a full understanding now of the difficulties of the case, and seeing the result of the extraordinary efforts which had been made in this operation, I contemplated, I frankly confess, another operation with dread and ill forebodings.

I determined, however, that I would not undertake another until I could devise some plan of securing the patient effectually in the right-angle position upon the knees, which I had had in contemplation for several years; and, if possible, to complete my new speculum, believing that no better case could be found to test its merits. Accordingly, I drew a plan of my *thoracic rest*, alluded to in the former part of this paper, and placed it in the hands of a carpenter, who had it ready for use in five or six days.

As to the speculum, this was not so easily completed, as it involved a radical change in my original plan, arising from a fundamental error in its construction, which I had not discovered until this particular juncture. An explanation of this change would necessarily require a description of the instrument and all the alterations made in it from the beginning, which would far exceed the limits assigned to these remarks in the outset.

On the 20th of November it was so near completed as to enable us to use it.

The patient was now placed in our new position, and thus secured upon the *thoracic rest*. The position was now found to be admirable, and the confinement of the patient perfect.

Chloroform was next administered, and our speculum, as here shown and described, was introduced and expanded to the fullest extent. A reference to the limit pointed out on a former page will give some idea of the enormous size of the vagina. In short, the dilatation of the vagina, regarding all the indications which we have pointed out, was most complete and satisfactory. The insignificant fistule which we had labored so hard to bring into view a few weeks before and failed, now showed itself in its fullest dimensions, steady and immovable, even in the very face of the most violent expulsive efforts of the patient from bearing down and vomiting, before which we stood almost powerless and helpless in the previous operation, with every assistant that could be employed.

I now viewed the parts of operative procedure, for the first time, with a feeling of certainty as to the result. At my leisure I began the operation, and quietly completed it by my ordinary method, without the aid of an assistant, further than to wash sponges and give chloroform.

In twenty-five minutes our patient was removed from the table and placed in bed, totally unconscious of what had been done. Ten minutes of this time, I should observe, were lost in consequence of a little hemorrhage which had to be controlled before introducing our sutures.

Thus was achieved, I conceive, the greatest triumph of our professional life.

The above operation was done in the presence of Drs. Thos. C. Finnell, Thomas S. Bahan, Joseph S. Crane, of New York, and Dr. Chabert, of Hoboken, all of whom expressed their entire satisfaction at the result.

To Dr. Finnell, I am under many obligations, for having so opportunely placed under my charge the above patient, so well adapted to the completion of our instrument. Without such an opportunity, our success might have been deferred many months longer. There are also due Dr. Chabert, many thanks for his kind attention to the patient during the after treatment.

The result in this case, however satisfactory it may be viewed in the important respects mentioned, merits additional interest, I think, from the fact that the instrument actually employed in the case, and from which these drawings have been made, was completed by my own hands in *gutta percha, sheet lead, and iron wire.* To Messrs. Geo. Tiemann & Co., 67 Chatham St., however, I am under great obligations as regards the foot, leverage, and legs of the instrument, and the many changes and alterations made from time to time, in order to reach this stage of completion. They placed at my disposal an experienced and finished workman, who made and put together almost every part of the instrument above named under my own supervision. Without this very great advantage, I never could have gone through with the work, even to this extent.

As regards the ultimate success of this instrument, from what we have seen thus far in its application, I think I can very confidently recommend it to the general practitioner, as well as the surgeon, as likely to give satisfaction in all cases where a speculum requires to be used.

That a diminution of the size of the instrument shown here will have to be made to suit the majority of cases, I am convinced. This is an extra large size. A medium size, I think, will cover four-fifths of all cases; one smaller size, and a larger one, such as here shown, covering the other fifth of the cases. In this last class, we include such cases as the one above detailed, and all cases with shortening of the vagina, resulting from injury of its walls or otherwise. As soon as we can determine properly the alterations necessary to be made in the proportions of this instrument, in order to reach the other two sizes, we will have them made.

The instrument, when completed in steel and electro-plated, as designed, will not, I am satisfied, exceed the weight of this, our original pattern, which is only eight ounces, being two ounces less than the ordinary lever speculum.

*Fifth Avenue Hotel.*

## Figures and Tables

**Fig.1 f1:**
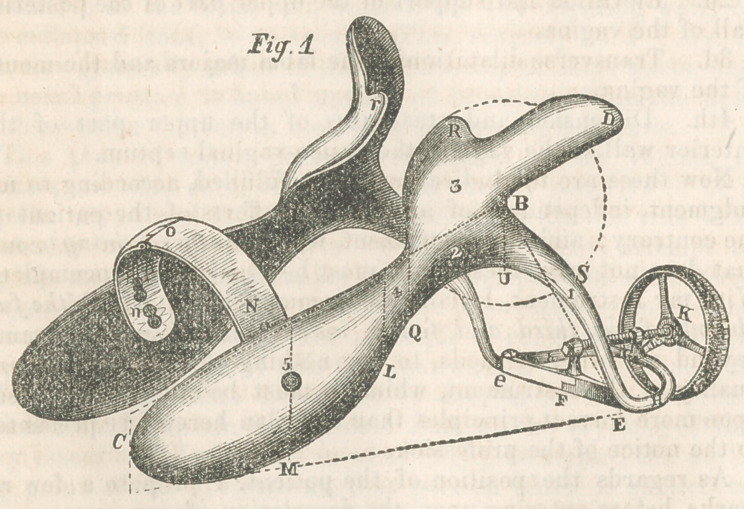


**Fig.2 f2:**
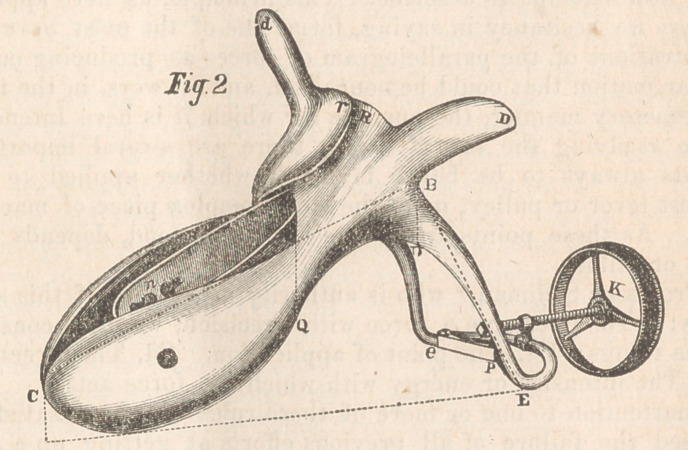


**Fig. 3. f3:**